# Balancing the Roles of Clinicians and Police in Separating Firearms from People in a Dangerous Mental Health Crisis: Legal Rules, Policy Tools, and Ethical Considerations

**DOI:** 10.1017/jme.2023.44

**Published:** 2023

**Authors:** Evan Vitiello, Kelly Roskam, Jeffrey Swanson

**Affiliations:** 1: UNC CHAPEN HILL, CHAPEL HILL, NC, USA; 2: JOHNS HOPKINS UNIVERSITY, BALTIMORE, MD, USA; 3: DUKE UNIVIERSITY, DURHAM, NC, USA

**Keywords:** Gun Control, Mental Health, Police

## Abstract

In COVID’s immediate wake, the 2020 death toll from a different enemy of the public’s health — gun violence — ticked up by 15 percent in the United States from the previous year. Meanwhile, the U.S. Supreme Court issued an opinion in *Caniglia v. Strom* that will allow people who have recently threatened suicide — with a gun — to keep unsecured guns in their home unless police take time to obtain a search warrant to remove them.

In a nation with a constitutionally protected individual right to bear arms, narrowly applicable legal rules constrain police interventions to separate firearms from putatively dangerous individuals. The Fourth Amendment to the United States Constitution protects people from unreasonable searches and seizures.^1^ Perhaps the most well-known exception allows for searches and seizures pursuant to a valid warrant; however, courts also recognize a number of exceptions that permit warrantless searches and seizures. Whether the threat of suicide satisfied a Fourth Amendment exception for “community caretaking” was under scrutiny in the recent U.S. Supreme Court case *Caniglia v. Strom*.^2^ The Court sided with appellant Edward Caniglia who challenged the warrantless police seizure of his firearm during an alleged psychiatric emergency as a violation of his Fourth Amendment right. Police officers may be empowered to enter a person’s home without a warrant and seize firearms, under the “exigent circumstances” exception to the Fourth Amendment’s warrant requirement, but “exigent circumstances” is a term that eludes a precise definition and, as applied, could allow some individuals in high-risk situations to retain their firearms because the police do not have time to obtain a warrant.

This Article discusses *Caniglia* and applies evidence from the research literature on clinical risk prediction to examine the problem of “exigency” in implementing legal interventions to prevent suicide by restricting access to lethal means. We argue that it is inherently difficult, if not impossible, to reliably predict suicide with enough temporal precision in every potentially urgent situation to meet the standard of “exigency” that Supreme Court case law seems to require for a warrantless firearm removal, while simultaneously ensuring the safety of the individuals of concern. We explore potential solutions to this problem.

We propose that an expanded use of extreme risk protection orders (ERPOs) could, in many cases, prevent the *Caniglia* situation from arising. In this regard, ERPOs could prevent suicides through earlier crisis intervention, especially if clinicians were to become more routinely involved in assessing suicide risk and initiating ERPOs.

According to the Centers for Disease Control and Prevention (CDC), as of 2020, suicides were the second leading cause of death for individuals aged 1-44, with homicides ranking third. Across all ages in 2020, suicide was the twelfth leading cause of death overall in the United States, with nearly twice as many suicides as homicides. Firearms are the most commonly used method for completed suicides, accounting for over 50% of suicides.

In general, we argue that law enforcement and behavioral health providers ideally should play coordinated and complementary roles in suicide threat response and risk assessment in order to prevent suicide deaths. But the challenge in preventing suicide is that it is inherently difficult to predict, even for behavioral health experts, and especially if time sensitivity is the goal to justify overriding legal protections for an intrusive intervention that infringes on a person’s rights. Pathways to suicide are determined by many factors that interact in complex ways, which are often unique to each individual case. Many of the risk factors for suicide are nonspecific — they apply to far more people who will not actually die from suicide than who will. Conversely, many people who *do* end their own lives did *not* appear in advance to be at immediate high risk. In this light, clinicians’ expert risk appraisals are bound to be imperfect, but if their judgements are guided by experience as well as evidence at hand, they may still inform and improve decisions about suicide threat response; using the best legal tools at their disposal will help.

We suggest that ERPOs — a time-limited civil restraining order to separate firearms from persons with behavioral indicators of risk — could, if used widely, effectively prevent suicide in some cases similar to *Caniglia*. Implementing the routine use of ERPOs by clinicians as petitioners, as some states have done, would help to bring this promising legal tool to scale.

Finally, we discuss some ethical considerations in a hypothetical case pertaining to the use of ERPOs by clinicians as a tool intended to separate firearms from persons at risk of suicide. We argue that ERPOs could be used more extensively by clinicians as well as law enforcement in legally permissive ways that are effective in preventing suicide, but also respectful of the Constitutional rights of gun owners and the dignity of persons who suffer from mental health disorders. Additionally, we suggest that ERPOs should be widely implemented in the wake of the passing of the Bipartisan Safer Communities Act of 2022, given the ability to effectively reduce gun violence including suicides.

## Suicide and Extreme Risk Protection Order (ERPO) Laws

According to the Centers for Disease Control and Prevention (CDC), as of 2020, suicides were the second leading cause of death for individuals aged 1-44, with homicides ranking third. Across all ages in 2020, suicide was the twelfth leading cause of death overall in the United States, with nearly twice as many suicides as homicides. Firearms are the most commonly used method for completed suicides, accounting for over 50% of suicides.^3^ Thus, among a variety of policy approaches to reduce suicide, means restriction — including or limiting access, to firearms is often cited as an effective strategy. A systematic review evaluating suicide prevention outlined multiple studies demonstrating that interventions to reduce access to firearms can lower suicide rates, including buyback programs.^4^ A relatively new mechanism to remove firearms from potentially dangerous individuals is an Extreme Risk Protection Order (ERPO).

As codified in a growing number of state laws throughout the United States, ERPO statutes provide a civil restraining order for law enforcement to temporarily remove firearms from a person who poses an imminent risk of injury to self or others, but who has not necessarily broken the law and would otherwise be allowed to possess firearms. In practice, this innovative legal tool, often called a “red flag law” in the popular lexicon and emerging commentary, is being applied in a range of different kinds of cases involving persons behaving dangerously with a firearm, or who might acquire a firearm.^5^

Animated by public concern over mass shootings in recent years, as well as the organizing efforts of national and local advocacy groups,^6^ 17 jurisdictions enacted ERPO laws since the Sandy Hook massacre in 2013, for a total of 19 states and the District of Columbia as of September 2022.^7^ An expert consensus model for ERPOs was first developed by the Consortium for Risk-Based Firearm Laws and originally called a “gun violence restraining order.”^8^ The underlying ERPO scheme was inspired by precursor risk-based gun removal laws in Connecticut and Indiana and relies on the accepted legal framework of a civil domestic violence restraining order in which family and household members may petition for protections, including firearm provisions. All 50 states and the District of Columbia have domestic violence protective order laws and the majority have an established process related to firearm removal.^9^

A typical ERPO law allows a law enforcement officer or family or household member to request a civil court order to remove a person’s firearms when there is probable cause to believe that the person poses a significant risk of bodily injury to self or others in the near future by possessing or acquiring a firearm. A few states add other categories of authorized petitioners, such as state’s attorneys, educators and clinicians.

In most ERPO statutes, the court may first issue an *ex parte* emergency extreme risk order authorizing law enforcement officers to immediately remove the respondent’s firearms, after giving the person an opportunity to voluntarily relinquish their firearms. The emergency order usually expires in 14 days following its issuance, and within that period the court must hold a hearing and the state must show typically by clear and convincing evidence that the respondent continues to pose a substantial risk of harm to self or others; if the state meets this burden, the judge may enter a ruling for an extended risk order, typically for a duration of up to 12 months. While the order remains active, the respondent is prohibited from possessing or purchasing firearms, but the person can retrieve the firearms upon expiration or termination of the order if they are not prohibited from purchasing or possessing firearms for any other reason.^10^

Connecticut (in 1999) and Indiana (in 2006) were the first two states to enact risk-based, temporary gun seizure laws, and the first empirical studies of the implementation and effectiveness of these types of laws were conducted in these two states. In both states, a death record study found that the population of individuals who were subject to gun removal had a base rate of suicide 30 to 40 times higher than in the general population. In both states, investigators estimated that for every 10 to 20 gun-removal actions, 1 life was saved by averting a gun suicide.^11^ More recent data from California, Washington, Colorado, Connecticut, Maryland, and Florida show that approximately 10 percent of ERPOs are being used in response to mass casualty-shooting threats.^12^

We turn to a recent U.S. Supreme Court case related to firearm seizure to prevent gun violence, while discussing the role that ERPOs may play in suicide risk mitigation.

## Caniglia v. Strom

On August 20, 2015, Edward Caniglia and his wife argued in their Cranston, Rhode Island home, escalating to the point that Mr. Caniglia threw his handgun on the dining table and stated, “why don’t you just shoot me and get me out of my misery.”^13^ His wife, rather than shooting him, opted to spend the night at a hotel after hiding the gun and magazine due to concerns for her husband’s state of mind. The next morning, after being unable to reach her husband, Mrs. Caniglia called police and requested they do a welfare check on her husband. When officers arrived at the home, Mr. Caniglia appeared to be calm, claimed he would never commit suicide, and when asked of his mental health told police it was none of their business. Still, Mr. Caniglia agreed to be transported to a local hospital for a psychiatric evaluation, he claims, on the condition that the officers would not take his firearms. After being evaluated by a physician, nurse, and social worker, he was discharged that same day, but not before the responding officers had removed his firearms and magazines believing that if the firearms remained in the home Mr. Caniglia, Mrs. Caniglia, and their neighbors could be in danger.^14^

Mr. Caniglia sued, claiming that law enforcement’s warrantless seizure of his firearms and requirement that he submit to a psychiatric evaluation violated his Fourth Amendment rights. He further claimed his Second Amendment right was violated by depriving him of his guns. The district court rejected Mr. Caniglia’s claims, relying on the community caretaking exception to the warrant requirement first articulated in the Supreme Court case *Cady v. Dombrowski* in the context of a warrantless search of a disabled vehicle’s trunk.^15^ The Court noted that the officers’ removal of his firearms was reasonable to protect the public. The district court further cited the importance of police welfare checks as part of the work of law enforcement.^16^ The First Circuit Appellate Court, also relying largely on *Cady*, affirmed the lower court’s holding, noting that “[t]here are widely varied circumstances, ranging from helping little children to cross busy streets to navigating the sometimes stormy seas of neighborhood disturbances, in which police officers demonstrate, over and over again, the importance of the roles that they play in preserving and protecting communities.” Additionally, the First Circuit described the murky overlap of community caretaking with exigent circumstances, in which the urgency of the situation requires officers to act in the face of insufficient time to obtain a warrant and the emergency aid exception, in which a person within the home has already sustained a serious injury or will do so in a matter of moments, but noted that the officers invoked neither the exigent circumstances nor the emergency aid exceptions.^17^

In a unanimous decision delivered by Justice Thomas, the U.S. Supreme Court vacated the lower courts’ decisions, remanded the case to the district court for further proceedings and refused to expand the community caretaking exception to warrantless seizures in a person’s home, distinguishing this circumstance from that in *Cady*, in which guns were seized during a search secondary to impoundment of a disabled vehicle. The Court differentiates the impermissibly broad interpretation of the community caretaking exception defendants urge be adopted with other narrower exceptions to the warrant requirement recognized by the Court in previous cases such as “when certain exigent circumstances exist, including the need to ‘render emergency assistance to an injured occupant or to protect an occupant from imminent injury.’” Caniglia did not raise the Second Amendment claim at the Supreme Court.^18^

Justice Alito, in his concurring opinion, discussed an important category of cases that could be viewed as involving community caretaking — the prevention of suicide, where police might immediately detain someone who is threatening suicide and transport them to a healthcare facility for evaluation. Although the Supreme Court has not opined on the short-term confinement of individuals who need further psychiatric assessment in emergent scenarios, Justice Alito noted that some states have enacted ERPO laws intended to prevent gun violence, whether it be suicide or harming others. The Court declined to address the constitutionality of these ERPO laws, though Justice Alito noted they may be challenged under the Fourth Amendment in the future.^19^

Chief Justice Roberts and Justice Kavanaugh, in separate concurring opinions, expressed assurances that police officers are permitted to assist people in need in their homes, including preventing violence, under the exigent circumstances doctrine.^20^ Justice Kavanaugh clarified that though many courts have relied on community caretaking to allow for warrantless entries into the home for the purpose of preventing suicide, he noted that this “issue is more labeling than substance.” Though the officers in *Caniglia* did not invoke exigency, Justice Kavanaugh felt compelled to describe the exigent circumstances exception and its applicability to the prevention of suicide. According to Justice Kavanaugh, the exigent circumstances exception “permit[s] warrantless entries when police officers have an objectively reasonable basis to believe that there is a current, ongoing crisis for which it is reasonable to act now … The officers do not need to show that the harm has already occurred or is mere moments away, because knowing that will often be difficult if not impossible in cases involving, for example, a person who is currently suicidal[.]” To illustrate this, Justice Kavanaugh poses the following hypothetical: a woman calls 911 and says that she is thinking about killing herself. That she has firearms in the home and she may as well die. Officers are dispatched to the woman’s home but she does not answer the door when they knock. According to Justice Kavanaugh, “of course” the officers may enter the home without a warrant.^21^

## Exigent and Imminent

Two legal concepts, exigency and imminence, are often used to define circumstances that may justify law enforcement’s immediate intervention to protect a person’s (or the public’s) safety. In *Caniglia v. Strom*, the U.S. Supreme Court outlines the “sacred” nature of the home as a castle in stark contrast to *Cady*’s impounded vehicle; however, the majority and all concurring opinions recognize that sometimes exigency demands that police enter the home.^22^

Though Justice Kavanaugh’s hypothetical above seems fairly straightforward, the moment when a mental health concern becomes a matter of exigency, justifying the state’s immediate intervention, often remains unclear. Further complicating the matter is when firearms are involved, raising questions about an individual’s Second Amendment rights. The Supreme Court, in *Kentucky v. King*, defined exigency as circumstances that “make the needs of law enforcement so compelling that [a] warrantless search is objectively reasonable under the Fourth Amendment.” The Court identified the following circumstances as exigent allowing for warrantless entry into a home: hot pursuit of a fleeing suspect, to prevent the imminent destruction of evidence, and “to render emergency assistance to an injured occupant or to protect an occupant from imminent injury.”^23^ How should police apply such a definition, practically speaking, in real situations where they are concerned about the safety of a person who has access to a gun? In the case of Mr. Caniglia, we have an armed man who states, “why don’t you just shoot me and get me out of my misery”^24^ and then later becomes unreachable to his wife. It is likely that many mental health professionals would have called a police welfare check in this scenario, as Mrs. Caniglia, in fact, did. Whether police welfare checks alone are effective interventions is debatable, however; there have been no standardized studies to evaluate their utility.^25^

To further highlight the challenging distinction of when a mental health emergency becomes an exigent circumstance, consider the 2014 mass shooting in Isla Vista, California. The family of the shooter, Elliott Rodger, had called for a police welfare check. The police came and talked to Mr. Rodger but concluded he did not meet strict criteria for involuntary detention and transport, importantly demonstrating the limitations of law enforcement conducting suicide risk assessments. However, had police been able to immediately enter Mr. Rodger’s dwelling and search for guns, they could have discovered and removed the weapons he later used to kill 6 people, and injure 14 others, before ending his own life. That the police should have had such authority became a key argument leading to the enactment of California’s Gun Violence Restraining Order, the first ERPO law to be adopted after the precursor laws in Connecticut and Indiana.

In summary, even if the police are called to a suicidal individual’s home and firearms are involved, the U.S. Supreme Court decision in *Caniglia* did not provide clarity on when exigent circumstances would justify removing access to lethal means without a judicial order. A concerned clinician could have made every effort to get psychiatric attention for a citizen, only for that individual to return to readily available access to lethal means. Relying on police welfare checks to meet this need is unlikely to be effective, especially if police are conducting their own suicide safety assessment and the Constitution (as the Supreme Court has interpreted the Second Amendment) provides citizens a broad right to retreat into their “castle” with firearms at hand. We argue that a more collaborative role between clinicians and expert clinical risk assessment in conjunction with police response could help to inform, and perhaps resolve, these kinds of situations.

How can ERPOs be scaled up? Recent federal legislation, the Bipartisan Safer Communities Act of 2022, includes an appropriation of $700 billion that states can use through grants from the Office of Justice Programs to improve implementation of ERPOs, on the condition that they have sufficient pre-deprivation and post-deprivation due process protections.^26^ One possibility for bringing ERPO implementation to scale would be through efforts to expand their use by clinicians. In every state with an ERPO law, clinicians can notify law enforcement about a safety concern involving a patient behaving dangerously with a firearm. However, in three jurisdictions — the District of Columbia, Hawaii, and Maryland — clinicians are authorized to petition the court directly for firearm removal under their ERPO law. Some scholars have argued that other states should follow suit and expand potential petitioners to include clinicians, noting the unique training and treating relationship allows the provider to assess the potential risk of a patient.^27^

One could imagine a scenario in which mental health clinicians are situated at the beginning of the cascade when firearms are removed. As discussed above, the Bipartisan Safer Communities Act will offer a large funding source for states to implement crisis interventions, including extreme risk protection orders. While this Act is largely geared toward public policy to reduce violence, there are clear implications and new resources for mental healthcare as it relates to individuals who pose a danger to themselves or others. This can place the mental health clinician in a position where they balance public and patient safety during crises that necessitate swift interventions, or the potential for imminent harms that demonstrate exigency.

## Risk Assessment

Mental health clinicians are often tasked with assessing patients for safety, including risk of suicide. Such assessments are fairly routine in psychiatric care and treatment follow-up, but they are especially called for at times when a patient may present with acute symptoms of psychopathology and a mental health crisis appears to be looming. Risk assessments are salient primarily to clinical management and therapeutic plans. A healthcare provider’s judgment that a patient poses a substantial risk of suicide can help determine the recommended setting, type, and intensity of treatment to mitigate the danger. But these appraisals of risk also carry legal consequences, insofar as they may be used to constrain an individual’s liberty. Examples include risk assessments to inform judicial decisions concerning involuntary civil commitment — that is, whether a person meets the common statutory criterion of “danger to self or others” — or whether to release a patient from a short-term involuntary psychiatric hold. Clinicians also can inform decisions to restore firearm rights through “relief from disabilities” proceedings in some states. In a variety of practice settings, clinicians are likely to encounter patients at elevated risk of suicide with access to lethal means — patients who would benefit from at least temporary removal of firearms as part of their safety planning.

Ideally, in theory, evidence from research and clinical studies of suicide risk could be used to inform law enforcement protocols to help officers better determine and document more accurately when a suicide concern is sufficiently “exigent” to merit removing a firearm without a search warrant. And there could be room for improvement in law enforcement practice in these difficult cases, perhaps through collaboration with behavioral health clinicians who have specific expertise in suicide risk assessment, as some communities have done. To effectively manage mental health crises, expanding the use of standardized screening instruments for suicide risk could be a part of the solution. The challenge, however, is that suicide screening instruments are a blunt tool when applied at the individual level; as already mentioned, they tend to identify suicide risk factors in many people who will never actually attempt suicide, while failing to detect elevated risk in others who do go on to end their own lives. Thus, the role of clinicians in weighing relevant risk factors remains crucial in suicide risk assessment rather than simple reliance on screening tools.

As clinicians try to weigh various risk factors during their suicide assessments, access to lethal means, namely firearms, is one of the most important. The American Psychiatric Association (APA) has published *Practice Guidelines for the Psychiatric Evaluation of Adults, Third Edition*, which includes a section on the assessment of suicide risk as well as the assessment of risk for aggressive behaviors. As part of that assessment, the APA recommends inquiry into the patient’s access of firearms.^28^ Similarly, the Department of Veterans Affairs (VA) and Department of Defense (DoD) have published *Clinical Practice Guideline for the Assessment and Management of Patients at Risk for Suicide*, which amongst numerous recommendations includes that special attention be given to access to firearms.^29^

Most of the suicide risk factors known to clinicians tend to be nonspecific — for example, certain symptoms of depression or suicide ideation, rather than overt threats. Determining when to intervene, and at what level of intensity, in situations involving indirect warning signs of suicide risk in individuals with particular characteristics is a difficult call. On the one hand, when private ownership and possession of a firearm is at stake, the consequence of a “false positive” could be depriving someone of a cherished right and perceived means of protection, when that person would not actually cause harm and has not broken the law. On the other hand, the consequence of a “false negative” error can be the tragic loss of a life.

To minimize both kinds of errors, optimizing the use of information from a suicide risk assessment involves a tradeoff between at least three estimated values: 1) the likelihood or probability that a suicide will occur if nothing is done to stop it; 2) the human cost — the sheer impact of individual and community loss associated with a suicide death; and 3) the burden of deprivation that a preventive action, such as gun removal, might impose on a person who would not actually die of suicide if the intervention were withheld.

Several self-report screening tools have been developed to help inform at least the first consideration in this balancing act — the absolute risk of suicide — but there are limitations to relying on these measures.^30^ For example, the Columbia-Suicide Severity Rating Scale (C-SSRS) was found to lack sensitivity for suicide risk after discharge from the Emergency Department (ED); most patients who died by suicide screened negative within 30 days and did not receive a psychiatric evaluation while in the ED. Additionally, most patients who screened positive for suicidal thinking died by a non-suicide cause.^31^ These findings highlight the challenge for identifying individuals at risk for suicide. To reiterate, many patients at genuine risk for suicide will not report feeling suicidal, and most individuals who do endorse suicidal thinking do not end their own lives.

The accuracy of suicide screening is limited, in part, due to heterogeneity in the population of individuals at risk for suicide, and the complex and diverse causal pathways to attempting and completing suicide, with access to firearms being a key risk factor concerning the completion of a suicide attempt. An individual’s ultimate risk of dying by suicide may be influenced by multiple biological, clinical, psychological, social, cultural, and environmental factors, such as method.^32^ A large meta-analysis evaluating 50 years of research revealed that suicide prediction is only slightly better than chance, and that predictive ability had not improved over the 50 years of studies on suicide.^33^ Thus, while there are numerous identified suicide risk factors, their predictive ability is lacking.

Given the limitations inherent in suicide risk prediction, emerging research has turned to predictive modeling that harnesses data within the electronic health record.^34^ In practice, clinicians consider a variety of risk factors and their interplay when assessing a patient’s risk for suicide, and they weigh these risk factors differently. For instance, psychiatrists and other behavioral health clinicians attribute more risk to patients who have a history of a prior suicide attempt as well as current expressed intent or desire to die with the presence of a suicide plan.^35^

To summarize, mental health clinicians are routinely placed in a position to assess a patient’s risk for suicide utilizing a variety of evidence-based tools. However, the predictive validity of these assessments is far from perfect, given that the pathway to suicide is complex and unique to each patient. It is inherently difficult, if not impossible, to operationally define the elastic concept of “exigency” in individual cases in a way that would systematically and accurately include or exclude future cases similar to *Caniglia*. Moreover, mental health clinicians have limited clinical interventions to adequately mitigate suicide risk in individuals who have ready access to lethal means such as firearms. Legal tools such as ERPOs, however, may offer a promising alternative.

We turn to a case and ethical analysis to imagine clinicians being situated at the beginning of firearm seizure, or namely, as petitioners for gun removal under an ERPO law.

## Ethical Analysis

The following is a hypothetical case which is not based directly on an identifiable patient. Mr. Doe is a 67-year-old White male in treatment with Dr. Smith for depression. The patient is a veteran and lives alone after the death of his partner six months ago. He was diagnosed with cancer last year and is undergoing curative treatment. However, Mr. Doe has struggled with depression despite therapy and antidepressant medication. Recently, after the death of his partner, the patient has expressed that he thinks of his death often and his desire to join his wife in the afterlife. Dr. Smith knows that Mr. Doe has two unsecured firearms in the home, as one of his hobbies is target practice. Mr. Doe has never been involuntarily hospitalized, and Dr. Smith does not believe that the patient’s current thoughts of death and being reunited with his wife constitute active suicidality. Nonetheless, Mr. Doe still represents someone at elevated risk for suicide, given numerous present risk factors for death by suicide including his age, race, gender, grief, medical illness, and access to firearms. Dr. Smith would like to respect Mr. Doe receiving care in the least restrictive environment possible, namely outpatient treatment as Mr. Doe is refusing to voluntarily present to the hospital, though the presence of multiple unsecured and loaded firearms is concerning.

Dr. Smith lives in a state that allows clinicians to petition for firearm removal under an ERPO. He thinks back to his training in medical ethics, remembering the concepts of autonomy, beneficence, nonmaleficence, and justice.^36^ Perhaps the first thought that comes to Dr. Smith’s consciousness is preventing a harm or bad outcome from occurring, consistent with the concept of *beneficence*, or the ethical principle of promoting the best interests of the individual or removing harms. Preventing suicide deaths is an identified public health goal, and as such, Dr. Smith sees the potential for great benefit in this encounter with a patient at risk for suicide — perhaps saving a life by removing access to lethal means. At the same time, Dr. Smith wonders if removing this patient’s legal access to firearms, even temporarily, might cause some harm, which would conflict with the principle of *nonmaleficence*. He fears that removing firearms from a veteran who enjoys collecting guns and engaging in target practice will damage the therapeutic alliance to such a degree that Mr. Doe will no longer engage in therapy or mental health treatment, while also missing his hobbies. He further fears that petitioning for firearm removal would represent a breach in confidentiality, as Dr. Smith is unclear if this would be a permissive disclosure to law enforcement and the courts akin to petitioning for involuntary commitment. Dr. Smith wonders if removing firearms through an ERPO would go against what Mr. Doe values, though also considers that most people value life and the ability to remain in the least restrictive setting, avoiding an inpatient hospitalization.

Let us also consider these principles from Mr. Doe’s perspective. We can imagine a situation, like that of Mr. Caniglia, in which police come to his house to conduct a welfare check due to concerns for his safety, and they remove the firearms without any warning. Imagine, in that scenario, that Mr. Doe’s clinician, Dr. Smith, was not consulted or involved, such that the police were ultimately left to evaluate suicide risk informally, with limited training and knowledge. As various media reports have shown us, there is the potential for the situation to be escalated by police presence and further harm to occur during that interaction. Perhaps the situation escalates, and Mr. Caniglia is taken by the police to a local emergency department for a psychiatric evaluation. We can imagine a number of events that occur in this cascade similar to the case of Mr. Caniglia, with Mr. Doe’s comparable feeling as though his autonomy and various rights are being violated.

Instead, if we consider Dr. Smith as the one who is situated as a petitioner for firearm removal, Mr. Doe will have a conversation with a mental health clinician regarding the reasoning and rationale behind the temporary seizure of the guns. Clinicians are trained in how to navigate these difficult situations in a therapeutic manner, a skill that the police — who are tasked with a variety of caretaking duties — do not typically have the time or training to master. During their clinical encounter prior to firearm removal, Dr. Smith and Mr. Doe have a discussion regarding the risk of unsecured and loaded guns combined with his various other risk factors for suicide. Dr. Smith explores Mr. Doe’s values and hopes for the future and options to treating his depression, while helping the patient understand lethal means reduction and strategies to prevent suicide. Rather than Mr. Doe being removed from the decision-making, he feels he had an opportunity to be involved and share his wishes. He may willingly engage in preventive measures to include temporarily surrendering his firearms to friends, storing them at the gun range, separating ammunition, or other ways to limit his access during this period of heightened risk. All these items are discussion points between Mr. Doe and his clinician surrounding strategies to prevent suicide deaths.

## Discussion

A public-health-focused, evidence-based approach to address America’s gun violence epidemic is constrained by the U.S. Supreme Court’s interpretation of the Second Amendment right to bear arms. The Court has recently expanded the potential exercise of this right, with two key decisions: *New York Pistol and Rifle Association vs. Bruen* ^37^ and *Caniglia v. Strom*.^38^ In *Bruen*, the Court held that it was unconstitutional for a state to require an applicant for a concealed carry license to show they have a good reason to need a gun. Rather, the Court recognized an individual right to carry a firearm in public — presumably including people who might be suicidal or impulsively angry.

Some experts believe that the long-term impact of *Bruen* will be to increase the number of at-risk individuals in the community with ready access to firearms, which in turn would increase the frequency of the very situation that the Court addressed in *Caniglia* — instances in which the police are called to intervene without delay when someone with a gun has threatened suicide or indicated a desire to die. Arguably, then, *Caniglia* makes it more difficult to solve the problem that *Bruen* has exacerbated.

For good measure, *Bruen* also requires lower appellate courts in the future to consider only Constitutional “text, history, and tradition” as the criteria for deciding Second Amendment challenges to states’ existing gun restrictions; this is likely to limit the opportunity for public health science to weigh in to help courts decide whether gun-related laws today serve a compelling Government interest (such as saving lives) and are narrowly tailored. We have argued that ERPO laws — by authorizing risk-based, time-limited firearm removal with due process protections — offer an important legal tool to prevent gun-related violence and suicide by temporarily removing firearms from persons who exhibit behavioral indicators of risk. ERPOs, if widely used, could mitigate the negative fallout of recent gun rights jurisprudence.

The typical standard for a judge to use when issuing an *ex parte* ERPO is probable cause to believe that the person of concern poses a significant risk of causing bodily injury to self or others in the near future. In the usual case, police officers play the leading role in petitioning for and serving an ERPO. However, in three jurisdictions, primary care clinicians and behavioral health providers are authorized to petition a court directly for an ERPO.

Expanding the role of clinicians in risk-based firearm removal actions through the use of ERPOs could reduce the need for police to wait before a potentially dangerous situation becomes exigent. In some situations, clinicians with training and experience in mental health crisis intervention might be better “caretakers” than police, who are trained in criminal law enforcement. In a therapeutic context with guidance from a trusted clinician, persons at risk might be more amenable to surrender their firearms pursuant to an ERPO — a time-limited civil restraining order — and without the need for heavy-handed police seizure. Thus, skillful clinicians empowered as ERPO petitioners may be better able to explain to their patients in crisis why removal of their guns is important for safety, obviating the need for encounters with law enforcement which has sadly been fatal for numerous individuals in a mental health emergency.

With the rise in mass shootings and school violence, public policy initiatives have often turned to “fixing mental health,” at least rhetorically, as a means to curb these incidents. The Bipartisan Safer Communities Act provides significant new funding opportunities to reduce gun violence in U.S. schools and communities both through gun safety measures and improving access to a continuum of mental health services, including appropriate crisis response interventions.^39^ In addition to non-psychiatric interventions, such as expansion of protections for victims of domestic violence or an enhanced background check process for potential gun owners under the age of 21, the Act features an expanded role for ERPOs.

Clinicians are authorized to initiate ERPOs in the District of Columbia, Maryland, and Hawaii. More states should consider adding and facilitating this option as part of clinicians’ toolkit for managing their patients’ risk of suicide. It would appropriately place the psychiatrist in a position they are already familiar with in civil commitment proceedings. In commitment hearings, clinicians play an important courtroom role, for example, offering empirical evidence on suicide risk factors, which in turn enables judges to make expert-informed decisions not solely relying on their knowledge of the law. To facilitate clinicians’ wider use of ERPOs under a permissive framework within these statutes, clinicians would ideally need to have immunity protection when they petition in good faith; Maryland statutes provide a model.

While public discourse on American gun violence often invokes images of mass shootings, such incidents account for a tiny fraction of gun deaths, while in reality suicides comprise the majority of firearm fatalities. Hence, we have focused here on the important intersection of mental health, suicide risk, and access to firearms. More than ever before, ERPO laws provide an important tool for stemming the tide of gun violence and suicide in the United States. The Bipartisan Safer Communities Act provides new resources to implement ERPOs widely, and state policy makers should seize the day.

While many clinicians may welcome this mechanism to remove firearms from patients at risk of causing harm, others could fear the implications of a further expansion of the treaters’ responsibilities. Also, diminishing reliance on police in some situations could be seen to threaten safety. Authorizing clinicians to initiate ERPOs could help to strike the right balance between risk and rights, caretaking, and protection, but it is important to carefully consider the ethical implications of this policy for patients’ privacy and trust, public safety, and clinicians’ duties. This expanded responsibility should be conceptualized similar to other reporting duties that mental health clinicians are already beholden to, such as child/elder abuse or the spectrum of *Tarasoff* duties that vary by state which invoke a duty to warn or protect potential victims of violence. However, in this construct of clinicians as ERPO petitioners, they would have a permissive ability to petition for firearm removal which would be statutorily protected in good faith circumstances. States should enact education and training for clinicians to ensure satisfactory knowledge of risk assessment, gun violence, and the construct of ERPOs.

## Conclusion

As ERPOs take hold and become more routine in situations similar to the *Caniglia* case, we have argued that clinicians — psychiatrists and other behavioral health specialists — can serve a vital and ethical purpose. Access to firearms remains one of the most robust risk factors for suicide in the United States, but there is good evidence to show that effective interventions to remove access to lethal means can prevent suicides. Behavioral health clinicians know this evidence well, and with an appropriate legal tool, they offer a critical opportunity to save lives.

While public discourse on American gun violence often invokes images of mass shootings, such incidents account for a tiny fraction of gun deaths, while in reality suicides comprise the majority of firearm fatalities. Hence, we have focused here on the important intersection of mental health, suicide risk, and access to firearms. More than ever before, ERPO laws provide an important tool for stemming the tide of gun violence and suicide in the United States. The Bipartisan Safer Communities Act provides new resources to implement ERPOs widely, and state policy makers should seize the day.

## Note

The authors have no conflicts to disclose.
